# Editorial: Follicular helper T cells in immunity and autoimmunity, volume II

**DOI:** 10.3389/fimmu.2026.1790802

**Published:** 2026-02-09

**Authors:** Shahram Salek-Ardakani, Georgia Fousteri, Maria Pia Cicalese

**Affiliations:** 1 Inhibrx Biosciences Inc, La Jolla, CA, United States; 2 Division of Immunology Transplantation and Infectious Diseases (DITID) Diabetes Research Institute (DRI), Istituto di Ricovero e Cura a Carattere Scientifico (IRCCS) San Raffaele Scientific Institute, Milan, Italy; 3 San Raffaele Telethon Institute for Gene Therapy (SR-Tiget), Istituto di Ricovero e Cura a Carattere Scientifico (IRCCS) San Raffaele Scientific Institute, Milan, Italy; 4 Pediatric Immunohematology and Bone Marrow Transplantation Unit, Istituto di Ricovero e Cura a Carattere Scientifico (IRCCS) San Raffaele Scientific Institute, Milan, Italy; 5 Università Vita-Salute San Raffaele, Milan, Italy

**Keywords:** autoimmunity, cancer, cytokine, follicular helper T cells (Tfh), follicular regulatory T cells (Tfr), germinal center, immunometabolism, peripheral helper Tcells (Tph)

## Introduction

Despite substantial progress in medicine, there remains an urgent need to address complex diseases through innovative immunological approaches. Over the past decade, follicular CD4^+^ T cell biology has expanded from a germinal center-restricted concept into a broader framework that now spans systemic autoimmunity, infection, and cancer. At its core, this area of study shows that these cells are not fixed types but rather flexible immune responders that conform to the body’s needs. Follicular helper T cells (Tfh), classically defined by CXCR5-driven follicular positioning and a transcriptional program centered on BCL6, with high ICOS and PD-1 expression, are essential architects of germinal center dynamics, affinity maturation, and durable humoral immunity (1, 2). In parallel, follicular regulatory T cells (Tfr), which integrate a regulatory lineage with follicular-homing features, have been recognized as critical regulators of this circuitry, restraining excessive B cell and Tfh activity to preserve tolerance (3, 4). More recently, the field has moved beyond canonical follicles to incorporate circulating Tfh states as biomarkers and peripheral helper T cell (Tph) populations as tissue-adapted engines of B cell help in inflamed sites (5, 6). Collectively, these advances have sharpened a unifying message: follicular CD4^+^ T cell programs are not static cell types, but adaptable immunologic states, continuously tuned by antigen burden, tissue damage, metabolic constraints, and microenvironmental context.

Volume II of this Research Topic assembles eight articles that reflect this maturation of the field. This volume asks three questions: how are T cell follicular programs wired, when are they constrained, and where do they become clinically actionable? Rather than simply reiterating that Tfh and Tfr “matter,” the contributions converge on addressing these key questions. Across infection, autoimmunity, tumor immunology, and Tfh-lineage malignancies, the central theme is that follicular CD4^+^ T cell biology is profoundly context-dependent. That context can be decoded mechanistically, tracked clinically, and potentially manipulated therapeutically.

## Damage sensing and temporal control of Tfh biology during infection

Two studies in this volume underscore that successful humoral immunity during infection is not entirely influenced by classic antigen-presenting and cytokine cues, but also by pathways that sense tissue stress and enforce developmental timing. This insight serves as a lens for vaccine design, showing how these damage-sensing pathways can be leveraged to amplify immune responses and improve clinical outcomes.

Russo et al. address a clinically meaningful paradox in pediatric RSV: severe disease can coincide with suboptimal antibody quality. By investigating moderate and severe RSV cohorts alongside healthy controls, they link clinical severity to a coordinated signature of impaired circulating Tfh biology, reduced cTfh frequencies, lower systemic IL-21 levels, and weaker neutralizing antibody responses targeting prefusion F (PreF). A key strength of the work is that it does not stop at association; instead, it identifies a plausible mechanistic axis connecting airway tissue damage to impaired helper function. The authors show that extracellular ATP is elevated in severe disease (in both systemic and respiratory compartments) and that cTfh cells exhibit increased expression of the purinergic receptor P2X7R, consistent with elevated responsiveness to a danger-associated metabolite. Functionally, P2X7R stimulation (using BzATP) triggers cTfh apoptosis, blunts proliferative capacity, and suppresses IL-21 production, effects that are at least partially reversed by pharmacologic antagonism. Together, the research suggests a coherent model in which excessive purinergic “danger” signaling during severe RSV acts as a brake on the very CD4^+^ helper compartment required for optimal humoral output, offering a new model for interpreting why antibody responses can be impaired in the setting of intense inflammation.

Supporting this “damage-sensing” framework, Zhu et al. focus on developmental control across time during chronic viral infection, using LCMV Clone 13 as a model of sustained antigenic stress. Their study positions THEMIS, best known as a T cell–specific adaptor involved in TCR signaling and selection, as a stage-specific regulator of CD4^+^ T cell fate that can both enable and constrain the Tfh program depending on timing. Early after infection, THEMIS supports the expansion and acquisition of Tfh differentiation features in virus-specific CD4^+^ T cells, consistent with a role in allowing follicular commitment under high inflammatory pressure. Strikingly, later in infection, the phenotype shifts: in competitive adoptive transfer settings, Themis-deficient CD4^+^ T cells ultimately generate stronger germinal center responses and higher antiviral IgG levels, accompanied by improved late viral control. Mechanistically, the authors connect this time-dependent switch to regulation of a progenitor-like TCF-1^+^ CD4^+^ pool (often conceptualized as a reservoir for durability), proposing that THEMIS preserves this progenitor state and thereby restrains excessive late skewing toward terminal follicular outcomes. Single-cell transcriptomic analysis at later time points supports a trajectory-level model in which THEMIS shapes the balance between progenitor maintenance and follicular differentiation under chronic antigen pressure. In aggregate, the work reframes chronic infection Tfh biology as a temporally gated process, one that may require early permissive signals but later restraint to optimize long-term immune architecture. A key next step will be determining whether pathways such as ATP–P2X7R signaling and THEMIS-dependent progenitor control are merely correlates of disease severity and chronicity, or whether they can be therapeutically modulated in a time- and tissue-restricted manner to improve antibody quality without exacerbating immunopathology or impairing viral control.

## Immunometabolism and signal integration drive heterogeneity in autoimmune Tfh-like programs

The autoimmunity-focused articles reveal another maturation of the field: the recognition that ‘Tfh-like’ expansion in disease is less informative than the quality of helper programs that emerge, including their metabolic dependencies and cytokine polarization. Helper quality can be defined by metrics such as autoantibody affinity and isotype skew, which provide concrete measures of how effectively these programs function. Specifying these metrics helps clarify the subsequent metabolic discussions, giving readers concrete results to associate with the functional capacity of the helper programs.

Bai et al. dissect functional heterogeneity between circulating Tfh-like (cTfh) and peripheral helper (Tph) populations in rheumatoid arthritis by explicitly linking effector phenotypes to distinct metabolic programs. They show that CXCR5^+^ cTfh cells correspond to a glycolysis-associated B-helper program, including higher expression of canonical transcriptional features associated with B cell help and follicular function, and propose that CXCL13–CXCR5 signaling helps sustain key glycolytic enzyme expression within this compartment. In contrast, CXCR5^-^ Tph cells show increased mitochondrial stress, elevated mitochondrial ROS, senescence-like features, and cytotoxic-leaning signatures, mechanistically connected to CCL2–CCR2 signaling. Significantly, the study moves beyond mapping to test perturbability: inhibition of glycolysis (2-DG) and scavenging of mitochondrial ROS (MitoQ) dampen inflammatory features in patient-derived cells and reduce disease severity in the collagen-induced arthritis model, with reduced accumulation of disease-associated pathogenic T cell states. The wider implication is that helper T cell pathogenicity in RA may be “metabolically maintained,” and that distinct chemokine-metabolic circuits support nonredundant helper programs that could be therapeutically targeted.

Verstegen et al. approach heterogeneity from a different angle, focusing on signal integration and functional plasticity during the acquisition of a Tfh-like phenotype. Using graded TCR-CD3 stimulation paradigms, they demonstrate that stronger signaling drives robust proliferation, IL-21 production, and the emergence of CXCR5+PD-1+ Tfh-like cells and additionally influences the cytokine identity within the IL-21+ compartment. Rather than treating IL-21 producers as a uniform population, they show that the balance between IL-4 and IFN-γ coexpression is strongly influenced by signal strength: weaker stimulation favors IL-4, while stronger stimulation biases toward IFN-γ within IL-21-producing cells. They then layer in extrinsic cytokine context, demonstrating that the cytokine environment further tunes these outputs, supporting the concept that Tfh-like effector behavior is a dynamically adjustable program integrating intrinsic antigen/TCR cues with extrinsic signals. The *in vivo* implications are conceptually important: antigen availability and persistence, together with local cytokine landscapes, may generate distinct “flavors” of Tfh-like help that differentially bias B cell outcomes. This raises a critical question for human disease: which Tfh-like endotypes are most relevant for patient stratification? Identifying these endotypes has the potential to clarify whether the metabolically and cytokine-polarized helper states described here represent stable, targetable ‘endotypes’ in patients. Moreover, whether intervention on glycolysis/mtROS or signal-strength-tuned polarization can selectively suppress pathogenic B cell help without losing protective humoral immunity and regulatory restraint remains an important unresolved issue.

## Follicular programs in tumor ecosystems and hematologic disease: from state shifts to lineage identity

Three articles expand follicular CD4^+^ T cell concepts within oncology and hematologic contexts, spanning immune-state remodeling in plasma cell malignancy, immunoregulation within lymphoma niches, and the clinicopathologic heterogeneity of Tfh-lineage lymphomas.

Zhang et al. provide an immunophenotyping view of helper and Tfh states across multiple myeloma disease stages, analyzing peripheral blood and bone marrow compartments across newly diagnosed, non-remission, and remission settings. A central observation is that PD-1/ICOS-defined Tfh subsets shift with treatment and remission: peripheral PD-1^+^ICOS^-^ Tfh populations decline from diagnosis toward remission, while PD-1^-^ICOS^+^ Tfh subsets rise following chemotherapy, consistent with remodeling away from checkpoint-skewed/dysfunction-associated phenotypes and toward a more activated or reconstituted helper landscape. In bone marrow, the authors highlight dynamic changes in Tfh2 and Tfh17 fractions, including differences in PD-1^+^ICOS^-^ Tfh17 between newly diagnosed and remission cohorts, and they explore whether peripheral measures may partially reflect marrow immunobiology. Notably, these shifts in PD-1/ICOS expression correlate with patient outcomes, as increases in PD-1^-^ICOS^+^ subsets are associated with better survival and lower relapse rates. While primarily associative, the research offers an organized framework for thinking about how CD4^+^ helper/Tfh states evolve over the course of disease and with therapy in MM, and it promotes a more thorough mechanistic link to tumor control and humoral features within the marrow tissue niche.

Rodriguez et al. focus on follicular lymphoma and address a key gap: the identity, origin, and functional role of Tfr cells within the malignant microenvironment. Through integrated cytometry, transcriptomic profiling, methylome analysis, and functional co-culture assays, they define FL-Tfr cells phenotypically (including CXCR5^+^CD25^hi ICOS^+^ features) and demonstrate that their transcriptional and epigenetic programs are closely aligned with classical Treg biology rather than representing a mere “Tfh-adjacent” state. Functionally, FL-Tfr suppress proliferation of autologous follicular helper and cytotoxic CD8^+^ T cells and exhibit suppressive capacity relevant to immune constraint within the FL ecosystem; the work also explores how suppression manifests relative to physiologic comparators. Notably, TCR analyses support lineage relationships, suggesting that at least a fraction of FL-Tfr shares precursor relationships with FL-Tfh, highlighting follicular-lineage plasticity within tumor niches. The study positions Tfr as an active immunoregulatory node in FL, an insight with direct relevance to therapeutic strategies aimed at remodeling lymphoma immune architecture.

Ma et al. extend the follicular theme into malignancy of the lineage itself by presenting an extensive, multicenter, retrospective clinicopathologic analysis of nodal Tfh cell lymphomas (nTFHLs), including a direct comparison of angioimmunoblastic type (nTFHL-AI), not otherwise specified (nTFHL-NOS), and follicular type (nTFHL-F). Their dataset reinforces that nTFHL is not a single clinicobiologic entity: subtypes differ meaningfully in clinical presentation, TFH-marker expression patterns, proliferative index, and outcomes, with nTFHL-NOS generally trending toward milder manifestations and nTFHL-F associated with inferior survival metrics. Beyond classification, the study evaluates real-world treatment patterns and reports that specific regimens outperform CHOP-like induction therapy in their cohort for response and survival endpoints. While the retrospective nature of the work and the need for deeper molecular stratification are appropriately acknowledged, the analysis offers an essential clinical anchor for a Research Topic otherwise rich in mechanistic immunology. It highlights the need for subtype-aware frameworks for prognosis and therapeutic decision-making. A major challenge moving forward is to link these phenotypic and lineage observations to causality, defining which Tfh/Tfr states meaningfully determine tumor control, immune escape, or therapy response, and identifying actionable nodes that remodel the microenvironment without triggering systemic immune dysregulation.

## Toward translational impact: the expanding Tfh–tumor research landscape

Finally, Lei et al. contribute a bibliometric analysis that maps global trends and emerging “hotspots” at the intersection of Tfh cells and tumors. By systematically surveying the literature over 2012–2024, they quantify publication growth, identify leading contributing countries, institutions, and authors, and use keyword/network analyses to infer shifts in scientific focus. The most informative aspect of the work is its depiction of conceptual progression, from earlier emphasis on expression-centric and B-cell-centric descriptors toward more recent concepts such as tumor microenvironment organization, immune infiltration states, and tumor-infiltrating immune cell ecosystems. In the context of the mechanistic and clinical papers assembled in Volume II, the bibliometric study functions as a meta-narrative: it highlights how the field is increasingly converging on tissue morphology, immune state organization (including tertiary lymphoid structures), and therapeutic relevance, precisely the terrain where follicular helper and regulatory programs are likely to matter most. As the field shifts toward microenvironmental architecture and immune infiltration states, a central open question is how best to standardize and integrate human tissue profiling (including TLS context, spatial organization, and longitudinal sampling) to generate reproducible biomarkers and mechanistically grounded targets for intervention. Furthermore, another unmet mechanistic question revealed by this publication trend is the identification of specific molecular pathways within the tumor microenvironment that could be manipulated to enhance or suppress Tfh and Tfr activity. This gap offers an important direction for future research, inviting scientists to explore specific interventions that might alter Tfh and Tfr functions in tumor settings, potentially revealing new avenues for clinical intervention.

## Conclusion

Together, the eight contributions in Volume II reinforce a field that is moving beyond the question of whether Tfh and Tfr are important, toward a more actionable understanding of what shapes them, how they evolve, and how they can be tracked or manipulated in human disease. A recurring message across infection, autoimmunity, and cancer is that follicular programs are governed by layers of control not always captured by canonical markers alone: damage-sensing pathways such as purinergic signaling, temporal regulators that balance progenitor maintenance against terminal differentiation, metabolic constraints that polarize helper states, and microenvironmental ecosystems that imprint regulatory function in tumors and lymphoma niches. As follicular T cell immunology continues to mature, an essential next step will be to integrate longitudinal human datasets with targeted perturbation, linking follicular CD4^+^ T cell states to antibody quality, immune pathology, and therapeutic response in ways that directly inform intervention design across infectious disease, autoimmunity, and oncology. Ultimately, the field will benefit from prospective, longitudinal human studies paired with perturbational models that can map follicular CD4^+^ T cell state to antibody quality and clinical outcome, clarifying when, where, and how to intervene on Tfh/Tfr circuits for durable therapeutic benefit.
